# Comparison of BMI changes in Japanese adults receiving face-to-face versus online counseling for specific health guidance: a noninferiority prospective observational study

**DOI:** 10.1093/joccuh/uiae026

**Published:** 2024-05-10

**Authors:** Satoru Kanamori, Kiyomi Tomiyama, Yasuo Haruyama

**Affiliations:** Graduate School of Public Health, Teikyo University, Tokyo, Japan; Department of Preventive Medicine and Public Health, Tokyo Medical University, Tokyo, Japan; Division of Health Support, Department Store Health Insurance Association, Tokyo, Japan; Integrated Research Faculty for Advanced Medical Sciences, Dokkyo Medical University School of Medicine, Tochigi, Japan

**Keywords:** internet, telemedicine, counseling, overweight, obesity, body mass index

## Abstract

**Objectives:**

This study aimed to evaluate the noninferiority of online counseling over face-to-face counseling for specific health guidance (SHG).

**Methods:**

This prospective observational study was conducted using specific health checkup (SHC) and SHG data of individuals with health insurance in Japan. We analyzed data from 1431 participants who met the inclusion criteria, including those who underwent online or face-to-face counseling between April 1, 2020 and March 31, 2021, and received an SHC in the following year but no earlier than 90 days after their first counseling session. Assessed variables comprised demographics, counseling methods, and SHC results, including baseline questionnaire findings and body mass index (BMI) at follow-up. We performed inverse probability of treatment weighting (IPTW) using propensity scores, with changes in BMI as the objective variable and the counseling method as the explanatory variable. We set the noninferiority margin to 0.175, based on a previous study.

**Results:**

The online and face-to-face counseling groups comprised 455 (31.8%) and 976 (68.2%) participants, respectively. The number of men and mean age were 214 (47.0%) and 49.9 years (SD: 6.9 years), respectively, in the online counseling group, and 491 (50.3%) and 51.1 years (SD: 7.6 years), respectively, in the face-to-face counseling group. IPTW using propensity scores revealed a regression coefficient of −0.014 (95% CI: −0.157 to 0.129) for the online group compared with the face-to-face group (*P* = .847). The CI was within the noninferiority margin.

**Conclusions:**

The effects of online counseling on BMI are likely noninferior to those of face-to-face counseling.

## Teaser

**What is already known on this topic**.

The coronavirus disease 2019 (COVID-19) pandemic has resulted in a swift surge in the use of online counseling as an alternative to traditional face-to-face counseling. Online counseling offers several benefits, such as heightened geographical inclusivity, facilitated participation for individuals facing travel constraints, and engagement with a broader spectrum of participants. Several previous studies have indicated that online counseling may be effective in improving overweight and obesity status.

**What this study adds**.

For specific health guidance in Japan, the potential effect of online counseling on BMI is expected to be comparable to that of face-to-face counseling.

**How this study might affect research, practice, or policy**.

As a practical implication, our results suggests that online counseling should be actively used when feasible. As a policy implication, our study indicates that the environment and system for online counseling should be improved, and that the skills of health professionals should be enhanced to develop effective online counseling.

## 1. Introduction

Overweight and obesity are on the rise worldwide,^1^ and are risk factors for diverse comorbidities (eg, type 2 diabetes, cancer, and cardiovascular diseases)^2^ and cause specific mortality (eg, vascular and diabetic mortality).^3^ Therefore, early screening and health guidance for overweight and obese individuals are necessary to reduce these risks.^4^

Until recently, most health guidance sessions have been conducted face-to-face. However, the COVID-19 pandemic has led to a rapid increase in online counseling instead of face-to-face counseling. A scoping review of the characteristics of online counseling compared with face-to-face counseling found that the advantages of online counseling include increased geographic diversity of participants, support for participation by those who cannot easily travel, and the ability to reach a wider range of participants.^5^ On the other hand, disadvantages have been shown to include reduced responses from the target population, difficulties in obtaining contextual information, and lower relationship satisfaction.

A systematic review of the effects of online counseling on obesity, lifestyle, and other health risks suggested that online counseling may be more effective in improving obesity than face-to-face counseling or providing information through personalized reports.^6^ However, this review only included 3 articles on obesity, all of which were of low quality in terms of research methods. An article not included in the aforementioned systematic review was a randomized controlled trial examining the effect of face-to-face versus online health counseling on weight loss in Japanese adults.^7^ It showed that online health counseling was not inferior to in-person counseling. However, attributes of the 155 participants other than age and sex were not described, which limited the external validity of the study. In addition, the outcome weights were obtained through interviews and information bias was present. Therefore, the number of participants who are overweight or obese for whom health guidance needs to be provided should be appropriately collected and examined using objective measures.

These studies indicate that online counseling may be effective in improving overweight and obesity. However, whether online counseling is inferior to face-to-face counseling in terms of weight loss effectiveness has not yet been clarified. Therefore, this study aimed to examine whether online counseling has a noninferior effect on body mass index (BMI) change compared with face-to-face counseling under specific health guidance (SHG) in Japan.

## 2. Materials and methods

### 2.1. Study design and setting

This was a prospective observational study using data from specific health checkups (SHCs) and SHG provided by the Department Store Health Insurance Association in Tokyo, Japan. The Department Store Health Insurance Association is a nationwide general health insurance association for small- and medium-sized retailers, including local department stores and supermarkets. As of April 2020, 288 establishments with 122 460 insured employees were enrolled in this health insurance program.

### 2.2. SHCs and SHG

SHCs and SHG mandate that all health care insurers assess the outcomes of health checkups and offer annual, systematic health guidance to individuals identified as requiring health improvement.^8^ One evaluation indicator for SHCs and SHGs is BMI. This guidance comes in 2 forms: active and motivational health guidance. A classification algorithm is employed to categorize the participants into groups, and the type of health guidance they receive is determined by their group constituents. Initially, the participants are classified based on obesity indicators, followed by the number of additional metabolic risk factors, smoking status, and age. Individuals who are currently undergoing pharmacological treatment for diabetes, dyslipidemia, or hypertension are not eligible for health guidance. Those included in any of the groups are considered to be at low risk and are provided with health information resources in the form of brochures. Systematic reviews and meta-analyses of the effectiveness of specific health counseling showed significantly lower changes in BMI in the group receiving either type of health counseling than in the control group.^9^

For both active and motivational support, at least 20 minutes of initial counseling (or at least 80 minutes of group counseling with 8 or fewer participants) is provided. Subsequently, only those eligible for active support will receive ongoing support for at least 3 months. These details have been described in previous literature^10^^,^^11^ examining the effectiveness of SHCs and SHG.

### 2.3. Participants

Health checkups in Japan are conducted based on the fiscal year (FY), which begins in April and ends in March of the following year. Of the 75 087 insured individuals enrolled in the Department Store Health Insurance Association who were eligible for an SHC in FY2020, 65 291 received an SHC in FY2020. Of the 11 780 participants who were eligible for active or motivational support for SHG, 783 received face-to-face counseling, 625 received online counseling, 131 received telephone counseling, and 10 241 did not receive counseling within FY2020. The participants who received telephone or no counseling were excluded from the analysis. We also assessed 324 and 204 participants who respectively received face-to-face and online counseling within FY2020 based on the results of their SHC in FY2019. In total, data from 1936 participants who received in-person or online counseling in FY2020 were evaluated.

**Figure 1:**
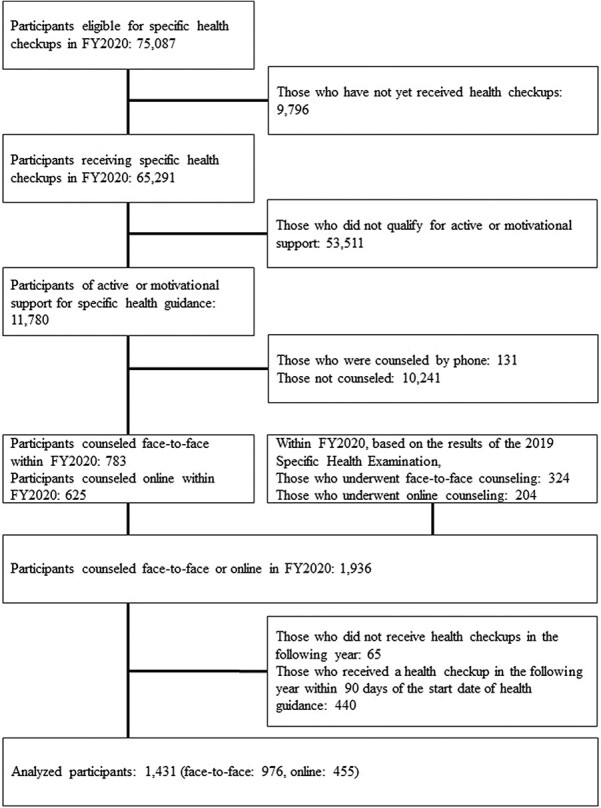
Flow chart of the study.

Of these 1936 participants, 1431 were included in the analysis, excluding 65 who did not receive a health checkup in the following year (including 6 who did not receive a health checkup due to retirement) and 440 who received SHG but received a health checkup in the following year less than 90 days after the start date of health guidance. Those who received a health checkup less than 90 days after the start of the health guidance were excluded because the guidance for the SHG stipulates^12^ that the first evaluation of the health guidance should be conducted 3 months after counseling. Of 1431 individuals, 270 had a combination of FY2019-2020, 5 had FY2019-2021, and 1156 had FY2020-2021 as the combination of their health examination results at the2 time points. A flowchart of the inclusion process is shown in Figure 1.

With reference to several previous studies, the sample size was calculated as follows using EZR (version 1.62 written by Kanda)^13^ software: the mean difference in BMI between the 2 groups was 0.61,^11^ the margin of noninferiority was 0.175 (see below for details), the SD was 3.4,^11^ the a error was .05 (1-tailed test), and the b error was .80. The sample size required for each group was calculated to be 232 participants. Therefore, the number of participants in this study exceeded the required sample size, thus ensuring a sufficient number of participants.

### 2.4. Measures

#### 2.4.1. Change in BMI

The objective variable was the change in BMI at the SHC at follow-up minus the baseline BMI. The BMI of each participant was rounded off to 1 decimal place.

#### 2.4.2. Health guidance methods

Occupational health nurses and dietitians from the Department Store Health Insurance Association conducted individual counseling and follow-ups based on SHG.^12^ One 30-minute counseling session was conducted with the motivational support participants. For the active support participants, a 30-minute counseling session was conducted, followed by 20-minute follow-up telephone counseling sessions 1 month, 2 months, and 3 months after the initial counseling session.

In the process of determining health guidance methods, the counseling method (online or face-to-face) was decided by the workplace to which the participant belonged; if the workplace or participant did not wish to be counseled, counseling was not conducted.

In cases where the health guidance methods were face-to-face, counseling was conducted at the store where the participant worked. In online cases, either Google Duo, FaceTime, or Zoom were used, depending on the participant’s preference.

Information on health guidance methods was extracted from counseling records maintained by the Department Store Health Insurance Association.

#### 2.4.3. Other variables

Other assessed variables were age, sex (male, female), headquarter location (Kanto region, non–Kanto region), company size (999 or less, 1000 or more), health guidance category (active support, motivational support), smoking (yes, no), exercise (yes, no), frequency of alcohol consumption (daily, less than daily), systolic blood pressure, aspartate aminotransferase (AST/GOT), alanine aminotransferase (ALT/GPT), γ-glutamyl transpeptidase (γ-GT/γ-GTP), triglycerides, high-density lipoprotein (HDL) cholesterol, low-density lipoprotein (LDL) cholesterol, and hemoglobin A_1c_ (HbA_1c_). The Kanto region comprises Ibaraki, Tochigi, Gunma, Saitama, Chiba, Tokyo, and Kanagawa prefectures. Because the headquarters of the Department Store Health Insurance Association are located in Tokyo and face-to-face visits to the Kanto region are relatively easy, the division of headquarters locations was divided between the Kanto and non-Kanto regions.

The SHC results at baseline were used to record all variables, except for the location of the head office and size of the company. The head office location and company size were extracted from the business establishment data in the core system of the Department Store Health Insurance Association.

### 2.5. Statistical analysis

To test the noninferiority of online counseling compared with face-to-face counseling, a margin of noninferiority was established. First, the effect of the active comparator was determined using the fixed-margin method.^14^ Based on the results of a meta-analysis examining the effect of SHG on BMI,^9^ the effect of the active comparator was set at −0.35. Then, based on a systematic review of methods for defining the noninferiority margin,^14^ we set the preserved fraction to 50%. Based on these values, the noninferiority margin was calculated by applying the formula from a previous study^15^ as follows: (1 − 0.50) × −(−0.35) = 0.175.

Two methods were used to test the robustness of the results to different analytical methods. The first method used a dataset with multiple imputations for missing values and an inverse probability of treatment weighting (IPTW) using propensity scores.^16^ Multiple logistic regression analysis was used to obtain a propensity score, with the method of health guidance as the objective variable and age, sex, headquarter location, company size, health guidance category, systolic blood pressure, AST (GOT), ALT (GPT), γ-GT (γ-GTP), triglycerides, HDL cholesterol, LDL cholesterol, HbA_1c_, smoking, exercise, and frequency of alcohol consumption at baseline as the explanatory variables. The average treatment effect was calculated based on the propensity scores and then used for the weighting. After weighting, standardized differences were calculated to ensure that participant characteristics were balanced between the face-to-face and online groups. Generalized linear models were then used to compare between the groups.

For missing values, a multiple imputation method was used to supplement each dataset, consisting of 2 groups of applicable individuals for comparison. All variables used in this study were subjected to multiple imputations and 20 datasets were created. These datasets were analyzed, and the results were integrated using Rubin’s method.^17^

Second, conventional multiple regression analysis was performed on the complete dataset, excluding cases with at least 1 missing item. In this analysis, the objective variable was the change in BMI, the explanatory variables were the method of health guidance, and the covariates were age, sex, location of headquarter, company size, health guidance category, systolic blood pressure, AST (GOT), ALT (GPT), g-GT (γ-GTP), triglycerides, HDL cholesterol, LDL cholesterol, HbA_1c_, smoking, exercise, and frequency of alcohol consumption at baseline.

In addition, a sensitivity analysis was performed excluding those whose baseline health examination results were obtained in 2019.

SPSS (version 28) was used for the analysis, and the 1-sided significance level was set at 2.5 %.

## 3. Results

Table 1 shows the participants’ characteristics at baseline and at follow-up using the health guidance method. The face-to-face group comprised 976 participants (68.2%), and the online group comprised 455 participants (31.8%). The number of companies to which the 1431 belonged was 56, the average number of applicable employees per company was 25.6, with the largest company comprising 205 employees and the smallest comprising 1 employee. Regarding the location of the head office, 71.0% of the face-to-face group and 58.5% of the online group were in the Kanto region. Regarding the classification of SHG, 47.7% of the face-to-face group and 58.2% of the online group were in the active support category. The mean values in triglycerides were 129.51 (SD: 100.95) in the face-to-face group and 146.94 (SD: 107.97) in the online group. A comparison of baseline and follow-up data showed significant decreases in smoking, BMI, systolic blood pressure,γ-GT, triglycerides, and LDL cholesterol levels in the face-to-face group. Significant increases were found for HDL cholesterol levels and HbA_1c_ percentages. In contrast, significant decreases in BMI, triglycerides, and LDL cholesterol levels were observed in the online group.

**Table 1:** Participants’ characteristics at baseline and follow-up according to the health guidance method.^a^

		**Face-to-face (*n* = 976)**							**Online (*n* = 455)**						
		**Baseline**			**Follow-up**				**Baseline**			**Follow-up**			
		***n***	**%**		***n***	**%**		***P***	***n***	**%**		***n***	**%**		***P***
**Sex**	Men	491	50.3		—	—		—	214	47.0		—	—		—
	Women	485	49.7		—	—			241	53.0		—	—		
**Head office location**	Kanto region	693	71.0		—	—		—	266	58.5		—	—		—
	Other than Kanto region	283	29.0		—	—			189	41.5		—	—		
**Company size (number of employees)**	999 or less	217	22.2		—	—		-	70	15.4		—	—		—
	1000 or more	759	77.8		—	—			385	84.6		—	—		
**Classification of specific health guidance**	Active support	466	47.7		319	32.7		—	265	58.2		175	38.5		—
	Motivational support	510	52.3		337	34.5			190	41.8		124	27.3		
	Other than the above	—	—		320	32.8			—	—		156	34.3		
**Smoking**	Yes	301	30.8		288	29.5		.011	137	30.1		137	30.1		1.00
	No	675	69.2		688	70.5			318	69.9		318	69.9		
**Exercising**	Yes	164	16.8		194	19.9		—	79	17.4		95	20.9		.198
	No	812	83.2		761	78.0			372	81.8		358	78.7		
	Missing	0	0.0		21	2.2			4	0.9		2	0.4		
**Alcohol consumption**	Everyday	207	21.2		198	20.3		.262	108	23.7		109	24		.498
	Less than everyday	769	78.8		778	79.7			344	75.6		344	75.6		
	Missing	0	0.0		0	0.0			3	0.7		2	0.4		
															
		***n***	**Mean**	**SD**	***n***	**Mean**	**SD**	***P***	***n***	**Mean**	**SD**	***n***	**Mean**	**SD**	***P***
**Age, y**		976	51.08	7.57	976	52.07	7.55	<.001	455	49.87	6.90	455	50.89	6.90	<.001
**BMI, kg/m** ^**2**^		976	27.61	3.55	976	27.42	3.79	<.001	455	27.68	3.69	455	27.48	3.73	<.001
**Systolic blood pressure, mmHg**		976	136.75	15.92	976	135.41	17.24	.004	455	133.39	15.00	455	132.12	16.43	.055
**AST (GOT), IU/L**		976	24.82	12.03	976	24.39	10.27	.203	455	26.55	13.97	455	25.64	12.09	.109
**ALT (GPT), IU/L**		976	29.98	27.27	976	28.89	21.07	.142	455	30.91	21.33	455	29.45	19.98	.082
**γ-GT (γ-GTP), IU/L**		976	41.94	45.50	976	40.04	40.45	.026	455	45.28	48.15	455	45.40	56.99	.947
**Triglyceride, mg/dL**		976	129.51	100.95	976	119.90	84.56	.001	455	146.94	107.97	455	126.88	90.09	<.001
**HDL-cholesterol, mg/dL**		976	60.70	16.44	976	61.95	16.55	<.001	455	59.68	15.51	455	60.37	14.51	.059
**LDL-cholesterol, mg/dL**		976	141.71	32.91	976	139.08	32.62	.001	455	141.50	34.38	455	137.07	33.88	<.001
**HbA**_**1c**_ **,** ^**b**^ **%**		778	5.66	0.58	778	5.69	0.66	.006	420	5.68	0.59	411	5.70	0.60	.423

Abbreviations: ALT (GPT), alanine aminotransferase; AST (GOT), aspartate aminotransferase; BMI, body mass index, ; γ-GT (γ-GTP), γ-glutamyl transpeptidase; HbA_1c_, hemoglobin A_1c_; HDL, high-density lipoprotein; LDL, low-density lipoprotein.

a A McNemar test was used for categorical variables; a paired *t*-test was used for continuous variables.

b For HbA_1c_, 776 participants were tested in the face-to face group and 397 in the online group.

Given that baseline data of the participants included a combination of those in FY2019 and those in FY2020, participants were divided into 2 groups and their characteristics were compared (Appendix 1). The results showed that the FY2020 group was significantly lower than the FY2019 group in terms of the percentage of companies headquartered in the Kanto region, the percentage of companies with >1000 employees, age, and in terms of HDL-cholesterol and LDL-cholesterol levels. Only BMI was inversely related.

Appendix 2 shows the participants’ characteristics at baseline after multiple imputations. The missing values for exercise, alcohol intake, and HbA_1c_ levels did not vary significantly after multiple imputations. Table 2 shows the participants’ characteristics after multiple imputations and IPTW. The standardized difference was less than 0.1 for all variables, confirming that the face-to-face and online groups were well balanced.

**Table 2:** Participant characteristics after multiple imputation and inverse probability weighting.

**Characteristic**	**Face-to-face**	**Online**	**Standardized difference**
	**%**	**%**	
**Sex, %**			0.04
**Men**	50.0	52.2	
**Women**	50.0	47.8	
**Head office location, %**			<0.01
**Kanto region**	67.4	67.1	
**Other than Kanto region**	32.6	32.9	
**Company size (number of employees), %**			0.03
**999 or less**	20.4	21.5	
**1000 or more**	79.6	78.5	
**Classification of specific health guidance, %**			0.01
**Active support**	51.2	51.8	
**Motivational support**	48.8	48.2	
**Smoking, %**			0.01
**Yes**	30.3	29.7	
**No**	69.7	70.3	
**Exercising, %**			<0.01
**Yes**	17.0	17.2	
**No**	83.0	82.8	
**Alcohol consumption, %**			0.02
**Everyday**	21.9	22.8	
**Less than everyday**	78.1	77.2	
			
**Age, mean, y**	50.70	50.64	<0.01
**Systolic blood pressure, mean, mmHg**	135.62	135.41	0.01
**AST (GOT), mean, IU/L**	25.72	25.32	0.03
**ALT(GPT), mean, IU/L**	30.95	30.32	0.02
**γ-GT (γ-GTP), mean, IU/L**	42.33	42.04	<0.01
**Triglyceride, mean, mg/dL**	135.11	135.86	<0.01
**HDL-cholesterol, mean, mg/dL**	60.38	60.08	0.02
**LDL-cholesterol, mean, mg/dL**	141.60	141.5	<0.01
**HbA**_**1c**_ **, mean, %**	5.65	5.65	0.01

Abbreviations: ALT (GPT), alanine aminotransferase; AST (GOT), aspartate aminotransferase; γ-GT (γ-GTP), γ-glutamyl transpeptidase; HbA_1c_, hemoglobin A_1c_; HDL, high-density lipoprotein; LDL, low-density lipoprotein.

Table 3 shows the association between the health guidance methods and changes in BMI. In the inverse probability weighted estimation method after multiple imputations, the regression coefficient for the online group compared with the face-to-face group was −0.014 (95% CI: −0.157 to 0.129) (*P* = .847). Furthermore, when multiple regression analysis was performed for complete cases (*n* = 1195) only, the regression coefficient for the online group was 0.008 (95% CI: −0.136 to 0.152) (*P* = .914). The upper CI did not exceed the noninferiority margin of 0.175 in either analysis. As a sensitivity analysis, an analysis was performed excluding the 2019 group of health screening data, which showed similar results (Appendix 3).

**Table 3:** Association between health guidance methods and changes in BMI.^a^

	**Inverse probability of treatment weighting (after multiple imputations), *n* = 1431**				**Multiple regression analysis (complete case analysis), *n* = 1195**		
	**β**	**95% CI**	***P***		**β**	**95% CI**	***P***
Face-to-face	reference				reference		
Online	−.014	−.157 to .129	.847		.008	−.136 to .152	.914

a Adjusted for age, sex, head office location, company size, health guidance category, systolic blood pressure, aspartate aminotransferase, alanine aminotransferase, γ-glutamyl transpeptidase, triglycerides, high-density lipoprotein cholesterol, low-density lipoprotein cholesterol, hemoglobin A_1c_, smoking, exercise, and frequency of drinking in multiple regression analysis (complete case analysis).

## 4. Discussion

The present study used longitudinal data to determine the effect of different counseling methods for SHG on changes in BMI and found that the amount of change in BMI in the online group was not inferior to that in the face-to-face group. These findings suggest that online counseling is as effective a method for reducing BMI as face-to-face counseling.

A randomized controlled trial examining the weight loss effects of face-to-face and online counseling methods for health guidance showed that online counseling is not inferior to face-to-face counseling.^7^ The results of the present study are consistent with those of this previous study. The limitations of the aforementioned study were that it was not limited to the target population of SHG and did not use objective outcome measures. In contrast, the present study addressed these issues and provides new findings. In addition, a systematic review and meta-analysis examining the effects of internet-based and face-to-face cognitive behavioral therapy on psychiatric and somatic disorders found no differences in their effects.^18^ The results of this study suggest that the same is true for weight loss.

Positive aspects of online counseling include avoiding the risk of infection, avoiding the social stigma of face-to-face exposure, and flexibility in scheduling.^8^ On the other hand, negative aspects include reduced responsiveness to the subject, difficulty in obtaining contextual information, lower satisfaction with the relationship, and difficulties in reaching a consensus.^5^ These positive and negative aspects may have offset each other so that there was little difference in the impact on BMI between online and face-to-face counseling.

A sensitivity analysis that excluded participants who received SHCs in FY2019 did not show significant differences from the results of the analysis that included them. There was no accumulation of data on poor health levels in either group regarding the baseline characteristics. Due to the system of SHCs in the Department Store Health Insurance Association, there is a time lag of several months between the date of the checkup and SHG implementation. In other words, those who received SHCs at the back end of FY2019 had no choice but to receive SHG in FY2020. In many cases, in the Department Store Health Insurance Association, the timing of health checkups is determined by the situation of the company for whom the individual works rather than by the individual’s own wishes. Individual health levels and literacy in information and communication technology may have little to do with the timing of health checkups. Hence, the results suggest that differences in the year of SHCs had little influence on BMI reductions due to differences in health guidance methods.

The result that online counseling is not inferior to face-to-face counseling in terms of health guidance for weight loss has several implications for the field. The first is to actively use online counseling when available. The implementation rate of SHG in Japan in FY2021 was 24.6%,^19^ which is insufficient. As online counseling can reduce constraints on time and access,^20^ the active use of online counseling may increase consultation rates. The second implication is to create an environment and system for conducting online counseling. In particular, health guidance providers need to have tools and communication environments for online counseling as well as the skills to conduct them effectively. Therefore, a system that supports the development of such environments is desirable.

This study has several limitations. First, there was a selection bias because the choice of counseling method for health guidance was dependent on the workplace and the participants. Health awareness and information and communication technology literacy on the part of employers and participants may have influenced the choice of counseling method. Although we were unable to address these factors in this study, we used as many countermeasures as possible by conducting IPTW methods using propensity scores based on the variables that we were able to address. Second, there was selection bias because only those who were counseled in both groups and underwent a health checkup in the following year were included in the study. A large number of participants were not counseled, which may indicate that the results were from participants who were more health conscious. Third, nearly a quarter of the 1936 eligible participants at baseline were excluded from the analysis, with 1431 (73.9%) participants remaining at follow-up. However, of the 505 excluded participants, 440 had received a health checkup in the following year, but less than 90 days after the start of the health guidance, and 6 had not received a health checkup at follow-up due to retirement. Therefore, those with low health awareness were not necessarily systematically excluded. Fourth, this study only included those insured by a single general health insurance association. Therefore, caution should be exercised when generalizing the results to other industries. Fifth, the period between the health guidance and the follow-up health checkup varied among the participants. It is difficult to say definitively at what point in time the results of this study represent a change in BMI. Sixth, the timing of the study coincided with the outbreak of a new type of coronavirus infection. It was also a time of major changes in working styles and daily life owing to the declaration of a state of emergency and encouragement for teleworking in Japan. A systematic review concerning the effect of obesity on risk factors in the first year of the COVID-19 epidemic revealed increased food and alcohol consumption, increased sedentary time, worsening depressive symptoms, and increased financial stress.^21^ Therefore, the captured situation may have differed from that during relatively normal periods.

## 5. Conclusions

The results of our examination of the impact of SHG on changes in BMI by counseling method among participants insured by the Department Store Health Insurance Association suggest that online counseling reduces BMI to the same extent as face-to-face counseling. Environments and systems must be improved to incorporate and promote online counseling.

## Supplementary Material

Web_Material_uiae026

## Data Availability

The data used in this investigation are not accessible in a communal database because they encompass information that may identify individuals or potentially sensitive patient data. In accordance with the ethical principles governing research in Japan, the Research Ethics Committee of Teikyo University imposed limitations on the distribution of the data collected during this study.
